# Global Burden of Human Brucellosis: A Systematic Review of Disease Frequency

**DOI:** 10.1371/journal.pntd.0001865

**Published:** 2012-10-25

**Authors:** Anna S. Dean, Lisa Crump, Helena Greter, Esther Schelling, Jakob Zinsstag

**Affiliations:** 1 Department of Epidemiology and Public Health, Swiss Tropical and Public Health Institute, Basel, Switzerland; 2 University of Basel, Basel, Switzerland; University of Oklahoma Health Sciences Center, United States of America

## Abstract

**Background:**

This report presents a systematic review of scientific literature published between 1990–2010 relating to the frequency of human brucellosis, commissioned by WHO. The objectives were to identify high quality disease incidence data to complement existing knowledge of the global disease burden and, ultimately, to contribute towards the calculation of a Disability-Adjusted Life Years (DALY) estimate for brucellosis.

**Methods/Principal Findings:**

Thirty three databases were searched, identifying 2,385 articles relating to human brucellosis. Based on strict screening criteria, 60 studies were selected for quality assessment, of which only 29 were of sufficient quality for data analysis. Data were only available from 15 countries in the regions of Northern Africa and Middle East, Western Europe, Central and South America, Sub-Saharan Africa, and Central Asia. Half of the studies presented incidence data, six of which were longitudinal prospective studies, and half presented seroprevalence data which were converted to incidence rates. Brucellosis incidence varied widely between, and within, countries. Although study biases cannot be ruled out, demographic, occupational, and socioeconomic factors likely play a role. Aggregated data at national or regional levels do not capture these complexities of disease dynamics and, consequently, at-risk populations or areas may be overlooked. In many brucellosis-endemic countries, health systems are weak and passively-acquired official data underestimate the true disease burden.

**Conclusions:**

High quality research is essential for an accurate assessment of disease burden, particularly in Eastern Europe, the Asia-Pacific, Central and South America and Africa where data are lacking. Providing formal epidemiological and statistical training to researchers is essential for improving study quality. An integrated approach to disease surveillance involving both human health and veterinary services would allow a better understanding of disease dynamics at the animal-human interface, as well as a more cost-effective utilisation of resources.

## Author Summary

Brucellosis is a bacterial disease transmitted to humans by consumption of infected, unpasteurised animal milk or through direct contact with infected animals, particularly aborted foetuses. The livestock production losses resulting from these abortions have a major economic impact on individuals and communities. Infected people often suffer from a chronic, debilitating illness. This systematic review of the incidence of human brucellosis is the first ever conducted. Using strict exclusion criteria, 28 scientific articles published between January 1990–June 2010 which included high quality data were identified. Half of these studies presented incidence data and half presented seroprevalence data which were converted to incidence rates. Data were only available from 15 countries in the regions of Northern Africa and Middle East, Western Europe, Central and South America, Sub-Saharan Africa and Central Asia. Brucellosis incidence varied widely between and within countries. Demographic, occupational and socioeconomic factors may play a role in these differences. In many brucellosis-endemic countries, health systems are weak, and official data are likely to underestimate the true disease burden. High quality research is needed, particularly from Eastern Europe, the Asia-Pacific, Central and South America and Africa. An integrated approach to disease surveillance involving both human health and veterinary services would allow a better understanding of the disease, as well as a more cost-effective utilisation of resources.

## Introduction

Brucellosis is one of the most common zoonotic infections globally [1], transmitted to humans through consumption of unpasteurised dairy products or through direct contact with infected animals, placentas or aborted foetuses. This bacterial disease causes a severely debilitating and disabling illness, with fever, sweating, fatigue, weight loss, headache, and joint pain persisting for weeks to months. Neurological complications, endocarditis and testicular or bone abscess formation can also occur [2]. Additionally, brucellosis has major economic ramifications due to time lost by patients from normal daily activities [2] and losses in animal production [3]. In a review of 76 diseases and syndromes of animals, brucellosis lies within the top 10 in terms of impact on impoverished people [4].

In 1992, the World Bank commissioned the original Global Burden of Disease (GBD) study, providing a comprehensive assessment of 107 diseases and injuries and 10 risk factors in eight major regions [5]. This review did not include any neglected tropical zoonoses. Such diseases often do not attract the interest of health researchers or sufficient resources for adequate control, yet they continue to impact significantly on human health and wellbeing, livestock productivity and local and national economies. There is a need for more accurate data relating to the burden of neglected zoonoses to facilitate more effective implementation of disease control interventions. In 2009, the Foodborne Disease Burden Epidemiology Reference Group (FERG) of the World Health Organization (WHO) commissioned a series of systematic reviews on the burden of neglected zoonotic diseases, with the aim of incorporating the findings into the overall global burden of disease assessments.

This report presents a systematic review of scientific literature published between 1990–June 2010 relating to the frequency of human brucellosis. The objectives of this review were to identify high quality disease incidence data to complement existing knowledge [6] of the global disease burden and, ultimately, to contribute towards the calculation of a Disability-Adjusted Life Years (DALY) estimate for brucellosis. A systematic review of scientific literature investigating the clinical manifestations of brucellosis is the subject of a companion paper.

## Methods

### Searching

Thirty three databases were searched for relevant articles using the search strings of both (brucellosis OR malta fever) and (brucellosis OR malta fever OR brucella melitensis OR brucella abortus) AND (symptom* OR sequelae* OR morbidity OR mortality OR transmission mode OR foodborne), with a publication limitation of 1990–30 June, 2010. The search term was adapted to the predominate language of the database. If a database did not allow the combining of Boolean operators, (18 of 33 databases), ‘brucellosis’ was used as the sole term.

Reference Manager bibliographic software was used to manage citations. Duplicate entries were identified by considering the author, the year of publication, the title of the article, and the volume, issue and page numbers of the source. In questionable cases, the abstract texts were compared.

### Selection

The articles were sorted by a team of four reviewers with a combined fluency in English, German, French, and Spanish. Articles in other languages were noted for future translation, pending resources.

All reports were classified into one of two categories, based on the abstracts:

Category 1: Relevant - articles related to human brucellosis infection in populations (i.e. disease frequency) or cases of human brucellosis (i.e. disease morbidity);

Category 2: Irrelevant - articles related to non-human brucellosis; articles addressing topics not related to the current review, such as genetics, laboratory diagnostic tests, experimental laboratory animal studies

The abstracts of studies belonging to Category 1 and meeting the following criteria for disease frequency were retained: published between 1990 and 30 June 2010, at least 100 study subjects drawn from the general population, prevalence or incidence data included and some information relating to diagnostic tests provided. The abstracts of studies meeting the following criteria for disease morbidity were also retained: published between 1990 and 30 June 2010, at least 10 study subjects, clinical symptoms/syndromes described and some information relating to diagnostic tests provided. The assessment and classification of morbidity articles will be the subject of a companion paper and will not be considered further here.

Articles for which the necessary data for classification could not be obtained were identified for possible future assessment, according to availability of resources. In general, non peer-reviewed or review articles, conference proceedings and book chapters were excluded.

### Validity Assessment

After applying the aforementioned screening steps, the full text of each selected article was retrieved for detailed analysis. Each article was reviewed by two or three reviewers, and classification discrepancies were resolved by discussion.

Frequency studies were classified as prevalence studies if they stated a specified study population and area and an outcome expressed as the proportion of the study population identified as brucellosis seropositive (%); or as incidence studies if they presented the time period of observation, information about the study population size and area, and an outcome expressed as the number of new brucellosis cases per population at risk per time period.

Articles were coded based on the following parameters:

1. **Study design.**
  - Longitudinal - clear start/end date with a study period of several months to years
  - Cross-sectional - a short study period of several weeks or, occasionally, several months
  - Routine data – data officially reported by health services or routine data recorded by a health facility or local authority
2. **Sampling methods.** The sampling approaches were defined in order of decreasing quality as: cluster sampling proportional to size, simple random cluster sampling, simple random sampling without clustering or non-random sampling. The method of case acquisition (active, passive) was also evaluated. Studies not meeting any of these classifications were coded as “other”.
3. **Study level.** The study area was categorised in decreasing order of quality as: national, provincial, district or sub-district level. Studies not meeting any of these classifications were coded as “other”.
4. **Diagnostic methods.** Tests were categorised in decreasing order of quality as:
  1. ELISA +/− Rose Bengal Test (RBT) or lateral flow assay only
  2. RBT only
  3. One of the following tests: microscopic agglutination test (MAT), complement fixation test (CFT), 2-Mercaptoethylamine test (2ME), standard tube agglutination test (STAT) of 1∶160 or greater dilution, Wright agglutination test (WAT) or Huddleson test.
  Studies diagnosing seropositives based on a STAT result of a dilution of less than 1∶160 were excluded.
5. **Study quality.** Studies were given an overall quality grade of 1, 2, or 3, as shown in Table 1. Quality 1 studies had well described study design and methods. Their sampling approaches and study level were highly ranked, e.g. active sampling by cluster sampling proportional to size or simple random cluster sampling approaches at the national or provincial level. The diagnostic methods were also highly ranked, such as ELISA, lateral flow assay or RBT. Quality 2 studies contained some weaknesses in their sampling approach and/or diagnostic methods. Although data were extracted from Quality 3 studies, they were not included in the final analysis, due to either a lack of information about the methods and approaches preventing adequate assessment of the quality of the study or obvious biases in study design and implementation.

**Table 1:** Grading of study quality based on study methodology criteria.

Methodological Criteria	Overall Study Quality		
	Quality 1	Quality 2	Quality 3
**Sampling approach**			
Cluster sampling proportional to size	✓	✓	
Simple random cluster sampling	✓	✓	✓
Simple random sampling without clustering		✓	✓
Non-random sampling			✓
**Case acquisition**			
Active	✓	✓	
Passive			✓
**Study level**			
National	✓	✓	
Provincial	✓	✓	✓
District	✓	✓	✓
Sub-district		✓	✓
**Diagnostic methods**			
ELISA with/without additional method	✓	✓	
Lateral flow assay with/without additional method	✓	✓	
RBT with additional method	✓	✓	✓
RBT only		✓	✓
MAT, CFT, STAT, WAT, 2ME, Huddleson alone or in combination		✓	✓

### Data Extraction

The following information was extracted from each article, and they were grouped according to geographic region, as identified by the GBD consortium:

*Seroprevalence studies*: study period, size of study population, seroprevalence as a percentage

*Incidence studies*: study period, size of reference population, number of cases, incidence rate

### Data Analysis

Seroprevalence data were multiplied by the duration of seropositivity, assumed to be 10.9 years [7], to determine the proportion of the general population seroconverting each year due to brucellosis exposure. Using a conservative estimate of 10% of seroconversions representing true clinical cases, these proportions were multiplied by 0.1 and converted to rates per 100,000 person-years for the general population.

### Additional Targeted Searching

Given that high quality data were also likely to be available through routine reporting systems in developed countries with strong public health systems, additional data sources were identified through a non-systematic, targeted search.

## Results

### Searching

Table 2 lists the databases searched and the number of articles identified for each. A total of 28,824 articles were identified, of which 59% were duplicates, leaving 11,000 original reports.

**Table 2:** Databases searched and number of hits.

Database	Website	No. hits
***Global databases***		
Medline	http://www.ncbi.nlm.nih.gov/sites/pubmed	6176
ISI Web of Science	http://isiwebofknowledge.com	3458
EMBASE	http://www.embase.com	4980
Popline	http://www.popline.org	55
CAB	http://www.cabdirect.org	3424
ProMed	http://www.promedmail.org	666
The Cochrane Library	http://www.thecochranelibrary.com	100
BIOLINE	http://www.bioline.org.br	37
WHOLIS	http://www.bireme.br	76
***Regional WHO databases***		
African Index Medicus	http://indexmedicus.afro.who.int	14
Index Medicus for the Eastern Mediterranean Region	http://www.emro.who.int/whalecom0/Library/Databases/wxis.exe/Library/Databases/iah/	526
Western Pacific Region Index Medicus	http://www.wprim.org/	96
Index Medicus for the South-East Asia Region	http://imsear.hellis.org/	247
Afro Library	http://afrolib.afro.who.int/	2
***Other regional databases***		
Health Information Locator	http://www.bireme.br	7
Institute of Tropical Medicine, Antwerp, Belgium	http://lib.itg.be:8000/webspirs/start.ws	122
King's Fund Information & Library Service	http://www.kingsfund.org.uk/library/	0
African Journals Online	http://ajol.info/	71
LILACS	http://www.bireme.br	538
MedCarib	http://www.bireme.br	9
REPIDISCA	http://www.bireme.br	29
PAHO	http://www.bireme.br	157
IBECS	http://www.bireme.br	148
CUIDEN	http://www.index-f.com/	17
Indian Medlars Center IndMed	http://indmed.nic.in/	84
KoreaMed	http://www.koreamed.org/SearchBasic.php	89
Japan Science and Technology Information Aggregator	http://www.jstage.jst.go.jp/search/?typej=on&typep=on&typer=on&search=1	137
Health Research and Development Information Network	http://www.herdin.ph/	0
Panteleimon	http://www.panteleimon.org/maine.php3	6
l'Ecole Nationale de la Santé Publique	http://test.bdsp.ehesp.fr/Base/	191
La Bibliotàgue de Santé Tropicale	http://www.santetropicale.com/resume/catalogue.asp	0
System for Information on Grey Literature in Europe	http://opensigle.inist.fr	474
Swiss Tropical and Public Health Institute, Human and Animal Health Unit, electronic departmental reference library		6906

### Flow of Selected Studies

**Figure 1:**
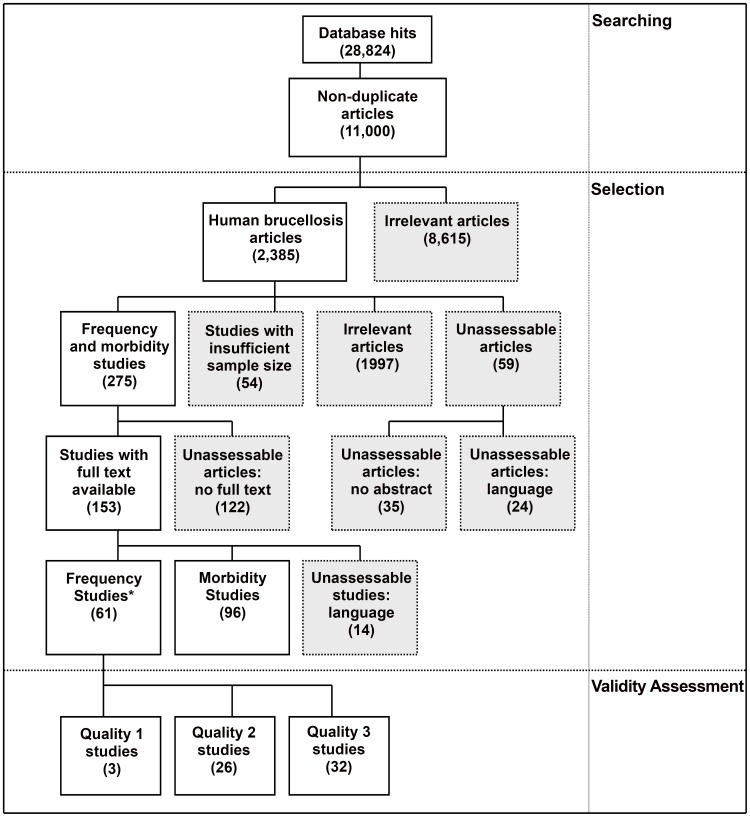
Flow of selected studies. *Some frequency studies were also classified as morbidity studies.

Figure 1 shows a detailed flow diagram of the selection of articles included in the systematic review. In total, 275 frequency and morbidity studies were selected, for which full text was available for 153. However, 14 of these were in languages in which the team was not competent (Croatian (6), Turkish (4), Korean (2), Persian (1), Mandarin(1)), leaving 61 frequency studies for quality assessment. Three were classified as Quality 1 and 26 as Quality 2. Thirty-two were excluded from further analysis as Quality 3, due to either a strong possibility of bias, a study population not representative of the general population, or a lack of adequate information to allow a proper assessment of study quality. Except for two articles in Spanish, all Quality 1 and Quality 2 studies were in English.

### Study Characteristics

Fifteen articles presented incidence data. Six of these were longitudinal prospective studies [8]–[13], with the remainder retrospectively reviewing data collected mainly in health centres [14]–[21]. Three incidence studies did not describe the diagnostic tests used but were included because they filled gaps in available data and had otherwise well-documented methods and results [15], [17], [20]. Seroprevalence data were presented in fourteen articles, from surveys conducted in communities [22]–[28] or from blood donor screening [29]–[32]. Due to a lack of data, several studies focusing on specific sub-groups of the general population where also included: two studies of nomadic communities [33], [34] and one of school children [35].

**Figure 2:**
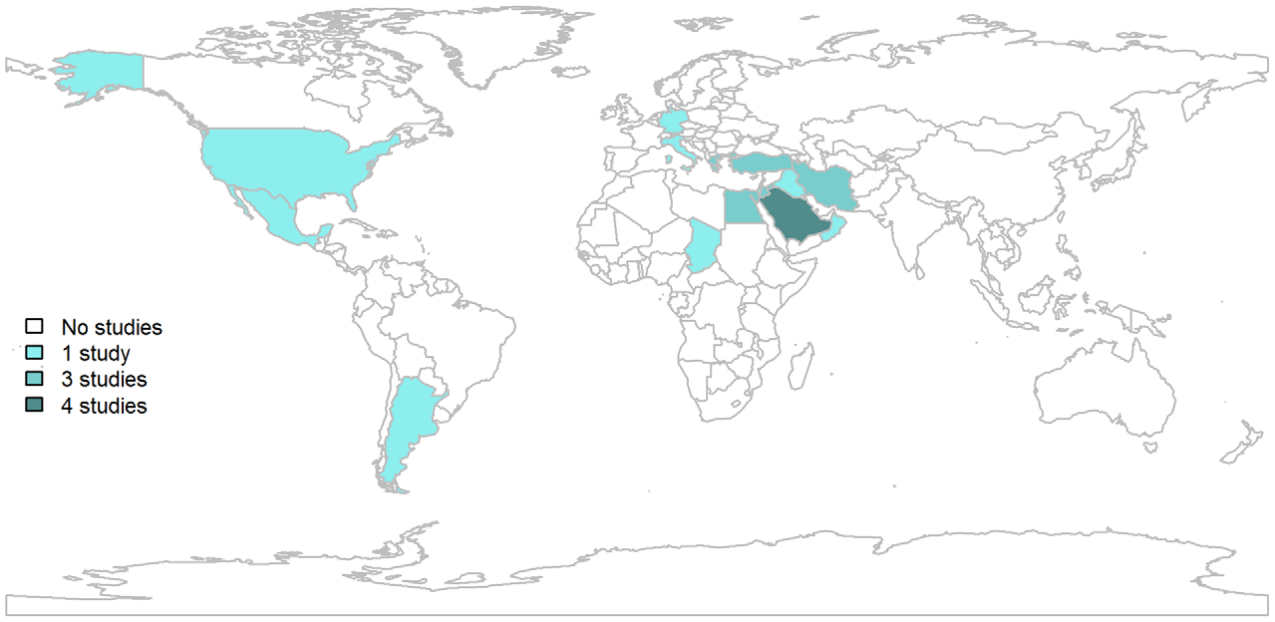
Geographical distribution of selected studies.

Studies of Quality 1 and 2 were only available for 15 countries from the following GBD geographic regions: Northern African and Middle East (17 studies) [8]–[11], [14], [16], [18], [19], [22], [25]–[27], [30], [32], [34]–[36], Western Europe (8 studies) [12],[13],[17],[20],[21],[23],[24],[28], South and Central America (2 studies) [29],[31], Sub-Saharan Africa (1 study) [33] and North America (1 study) [15], as shown in Figure 2. One additional Quality 1 seroprevalence study from Central Asia [7] was identified through targeted non-systematic searching which, although not fulfilling the publication date criteria of the systematic review, was included in data analysis because it provided otherwise missing data.

Study characteristics of Quality 1 and 2 seroprevalence and incidence articles are provided as Supplementary Information (Tables S1 and S2), grouped by GBD geographic region. Confidence intervals for seroprevalence estimates were only provided in four articles [7],[22],[25],[33]. The normal approximation to the binomial was used to calculate confidence intervals for the prevalence estimates of the remaining articles. Although some studies used a cluster-based sampling approach, they did not present adequate information for the calculation of adjusted confidence intervals.

### Data Analysis

Table 3 shows the incidence of brucellosis in the general population per 100,000 person-years by country, including rates directly reported as well as those calculated from seroprevalences. The studies are classified as containing data from the national and/or sub-national level. Where incidence rates were reported for several years, only the most recent data are provided.

**Table 3:** Brucellosis incidence by country (cases per 100,000 person-years).

Country	Study level	Incidence per 100,000 per year
**North Africa and Middle East**		
Egypt	Sub-national	0.28–70.00
Iraq	Sub-national	52.29–268.81
Iran	Sub-national○	0.73–141.60
Jordan	National	25.70–130.00
Oman	Sub-national*	11.01
Palestine	Sub-national	8.00
Saudi Arabia	National	137.61
	Sub-national	6.00–149.54
Turkey	Sub-national	11.93–49.54
**Sub-Saharan Africa**		
Chad	Sub-national+	34.86
**Western Europe**		
Germany	National	0.03
Greece	Sub-national	4.00–32.49
Italy	National	1.40
**Central Asia**		
Kyrgyzstan	National	88.00
**Central and Southern Latin America**		
Argentina	Sub-national	12.84
Mexico	Sub-national	25.69
**North America**		
USA	Sub-national	0.02–0.09

○ includes one study of a nomadic community.

* children only.

+ nomadic community.

A wide variation in reported brucellosis incidence is evident regionally, as well as within countries. In the North Africa and Middle East region, for example, incidences calculated from a seroprevalence study in Iraq ranged from 52.3 cases per 100,000 person-years in a rural area to 268.8 cases per 100,000 person-years in a semi-rural area [25]. In Egypt, two prospective incidence studies incorporating a surveillance system for acute febrile illness in different rural areas provided rates of 18 [8] and 70 [9] cases per 1000,000 person-years. Only 5.7% of these cases were detected through passive hospital-based surveillance [9].

Incidence rates in Western Europe and North America were generally much lower than in other regions, although some within-country variation was still evident. In Greece, for example, respective rates of 4 [20] and 32 cases [13] per 100,000 person-years were reported in western and central areas. The study in western Greece also identified that one quarter of these cases, although diagnosed in health facilities, were not officially reported to the provincial public health department. Although rates in the USA were very low, counties within 100 km of the Mexican border had a higher disease incidence (0.18 compared to 0.02) than those in non-border states [15].

### Additional Targeted Searching

Surveillance data from the European Food Safety Authority were obtained, giving an overall incidence for the European Union of 0.08 cases per 100,000 person-years, three quarters of which were reported by Greece, Spain, and Portugal [37]. Global data obtained non-systematically from various sources including health ministries, international organisations and scientific articles has been previously summarised according to continent and country by Pappas, and a global map was produced [6].

## Discussion

The epidemiology of human brucellosis evolved over the previous 15 years, as a result of socioeconomic factors, improved surveillance systems, animal-based control programs and international tourism [6]. Additionally, political changes have influenced disease epidemiology, with brucellosis emerging as a major human health problem in countries of the former Soviet Union following its dissolution in 1991 [3].

The current review complements previous assessments of brucellosis disease burden [6] by presenting epidemiological data from scientific studies published between 1990–June 2010 which have been quality screened according to strict criteria. There is an obvious lack of high quality scientific data relating to brucellosis incidence globally, with the majority of data coming from the North Africa and Middle East region. Major gaps exist for Eastern Europe and the Asia-Pacific, both of which had no available data, as well as for Central and South America (only two studies) and Africa (excluding Egypt, only one study). One of the major factors limiting the usefulness of the identified studies was the lack of clearly described methods, particularly in relation to sampling approaches and case definitions. For many studies, it was not possible to assess whether the study had been conducted in such a way to minimise the risk of bias, resulting in the exclusion of data that may have been of acceptable quality.

Brucellosis incidence varies widely not only between countries but also within countries. Although it is not possible to rule out study biases as potential causes of these differences, rates differing by five times in one study in Iraq [25] or by four times in similarly designed studies in Egypt [8],[9] suggest that demographic, occupational, and socioeconomic factors may play a role. Aggregated data at national or regional levels cannot capture these complexities of disease dynamics and, consequently, at-risk populations or areas may be overlooked.

A lower disease incidence is seen in developed countries when compared to low and middle income countries. However, brucellosis still targets specific sub-groups of these populations, such as Turkish immigrants in Germany [21] or Hispanic communities of low socioeconomic status in the USA [15]. Brucellosis clearly remains a disease of public health importance even in developed countries.

Although grey literature can provide high quality data, data from well designed scientific studies are preferred. Passively acquired national data in many brucellosis-endemic countries are likely to underestimate the true disease burden. In an Egyptian study incorporating an active acute febrile illness surveillance system to identify and confirm suspected cases, brucellosis incidence in the study area was 70 cases per 100,000 person-years. Only 5.7% of these cases were identified through hospital-based surveillance, from which the incidence rate would be calculated as 3.8 cases per 100,000 person-years using a case definition based on laboratory confirmation or 6 cases per 100,000 person-years using a clinical definition. Reliance on routine hospital-based incidence data would have, therefore, underestimated incidence by 12–18 times [9]. Official data from the Ministry of Health provided an incidence rate of only 0.3 cases per 100,000 person-years [6].

Such underestimations of disease incidence could relate to barriers to accessing health care or to case mismanagement and misdiagnosis. A retrospective review of hospital records in western Greece identified an additional source of error in official passively acquired data, with a brucellosis under-reporting rate from hospitals to the public health department of 26% [20]. Consequently, one quarter of cases diagnosed through the hospital system were not included in the official government data. Indeed, incidence rates identified in studies conducted between 1999–2005 in different regions of Greece ranged from 4–32.5 cases per 100,000 person-years, whereas aggregated data published by the European Food Safety provided a national incidence of just 0.9 cases per 100,000 person-years in 2009 [37].

### Research Agenda

Strengthening public health systems would improve the quality of data captured through routine reporting. In many brucellosis endemic countries, however, health systems are weak and high quality research is needed. Brucellosis incidence and prevalence studies are notably lacking from Eastern Europe, the Asia-Pacific, Central and South America and Sub-Saharan Africa. Researchers must have an adequate foundation in the principles of epidemiology and biostatistics to ensure that their studies are designed, implemented, and analysed in a manner which minimises bias and maximises the usefulness of the data.

An integrated approach to disease surveillance involving both human health and veterinary services would allow a better understanding of disease dynamics at the animal-human interface, as well as a more cost-effective utilisation of resources [38],[39]. A checklist of requirements for representative seroprevalence and incidence studies is given in Table 4.

**Table 4:** Key considerations for representative brucellosis seroprevalence and incidence studies.

	Seroprevalence	Incidence
**Study population**		
Defined study zone, geographically or in terms of an administrative unit	✓	✓
Clear inclusion and exclusion criteria	✓	✓
Study population representative of general population, not high risk groups alone	✓	✓
Ongoing community education campaigns to raise disease awareness	✓	✓
**Sampling**		
Sample size based on appropriate calculation, ideally including clustering of individuals	✓	
Random sampling strategy, ideally using probability proportional to size	✓	
Active surveillance system in health centres and/or in communities at household level		✓
Multidisciplinary study team to investigate disease dynamics at animal-human interface	✓	✓
**Diagnostic testing**		
Clearly described testing methods, including details of manufacturer or developer	✓	✓
Concise serological and clinical case definitions	✓	✓
ELISA, lateral flow or RBT preferred testing methods	✓	✓
**Reporting**		
STROBE checklist followed [40]	✓	✓
Consideration of test performance in the analysis of results	✓	✓
Consideration of sensitivity of the surveillance system in the analysis of results		✓

### Limitations

Although test performance was considered in the initial ranking of each article and, thus, influenced study inclusion in the review, the individual results of each study were not adjusted for test performance. This is because test performance can vary significantly according to test manufacturer or laboratory protocol, and such detailed information was not available.

The calculation of incidence rates from seroprevalence studies is based on two assumptions. The assumed duration of seropositivity of 10.9 years has been determined mathematically using Vensim software [7]. The assumption that 10% seroconversions represent true clinical cases is conservative and is likely to underestimate the true burden of disease.

Studies for which a title or abstract was not published in a language using the Latin alphabet, such as those published only in Chinese characters or Arabic script, may not have been identified during the original database search. Of the foreign language studies that were identified, those published in languages in which the team was not competent were excluded from the analysis. It is possible that some of these studies contained data that could have contributed to this global assessment of brucellosis frequency. Additionally, although studies in English were independently reviewed by three team members, this was not always possible for studies reviewed in other languages (German, French, Spanish).

### Conclusion

This systematic review adds to the understanding of the global burden of brucellosis by identifying high quality data from scientific studies according to strict screening criteria. Disease incidence varied significantly within regions and within countries. Aggregated data do not capture the complexities of disease dynamics and at-risk populations may be overlooked. As many brucellosis-endemic countries do not have strong health systems, passively acquired official data likely underestimate the true burden. The brucellosis research agenda should focus on designing and implementing high quality studies to investigate disease seroprevalence and/or incidence, particularly in Eastern Europe, the Asia-Pacific, Central and South America and Africa.

## Supporting Information

Table S1 **Selected brucellosis seroprevalence studies by region.**
(DOC)

Table S2 **Selected brucellosis incidence studies by region.**
(DOC)

Checklist S1 **PRISMA checklist.**
(DOC)
