# Supplementary material for: Global Burden of Human Brucellosis: A Systematic Review of Disease Frequency
Source: PLoS Negl Trop Dis. 2012 Oct 25;6(10):e1865. doi: 10.1371/journal.pntd.0001865 (PMC3493380; doi:10.1371/journal.pntd.0001865)
Supplement: Table S1 — Selected brucellosis seroprevalence studies by region. (DOC) [file pntd.0001865.s001.doc]

**Table S1: Selected brucellosis seroprevalence studies by region**

| **Country,**  **Year(s) of study** | **Author** | **Study design** | **Study level** | **Study population** | **Diagnostic tests** | **Seroprevalence (%), (95% CI)** | **Number of subjects** |
| --- | --- | --- | --- | --- | --- | --- | --- |
| **North Africa and Middle East** | | | | | | | |
| Egypt,  2003 | El Sherbini  [22] | Cross-sectional | Sub-district | General population | STAT | 0.03 (0-3) | 616 |
| Iran,  2004 | Alavi  [34] | Cross-sectional | District | Nomadic community | RBT; sequential testing of all positives with WAT; sequential testing of all positives to both tests with 2ME | 8.0 (7.1-8.9) (RBT);  7.9 (7.0-8.8) (RBT + Wright);  6.3 (5.5-7.1) (RBT + Wright + 2ME) | 3,594 |
| Iran, Unknown | Khorasgani  [30] | Unknown | Provincial | Healthy blood donors | RBT; sequential testing of all positives with STAT | 0.08 (0-0.1) (RBT);  0.06 (0-0.1) (RBT + STAT) | 10,500 |
| Iraq, Unknown | Yacoub  [25] | Cross-sectional | District | General population: rural | RBT | 5.7 (3.5-7.8) | 439 |
| Iraq, Unknown | Yacoub  [25] | Cross-sectional | District | General population: urban | RBT | 12.2 (9.1-15.3) | 435 |
| Iraq, Unknown | Yacoub  [25] | Cross-sectional | District | General population: semi-rural | RBT | 29.3 (25.1-33.3) | 465 |
| Jordan, 1996-1998 | Al-Ani  [32] | Longitudinal | National | Healthy blood donors and healthy people undergoing routine health exams | MAT | 4.1 (2.7-5.5) | 800 |
| **Country,**  **Year(s) of study** | **Author** | **Study design** | **Study level** | **Study population** | **Diagnostic tests** | **Seroprevalence (%), (95% CI)** | **Number of subjects** |
| Oman, Unknown | Idris  [35] | Cross-sectional | District | School children | MAT, followed by testing of positives by STAT | 1.2 (0.1-2.3) | 373 |
| Saudi Arabia, 1989 | Alballa  [27] | Cross-sectional | Provincial | General population | MAT, followed by STAT of positives | 16.3 (15.3-17.3) | 4794 |
| Saudi Arabia, 1997 | Al Sekait  [26] | Cross-sectional | National | General population | CFT | 15 (14.5-15.5) | 23,613 |
| **Sub-Saharan Africa** | | | | | | | |
| Chad, 1999-2000 | Schelling  [33] | Cross-sectional | Provincial | Nomadic community | Parallel ELISA + RBT | 3.8 (2-5) | 860 |
| **Western Europe** | | | | | | | |
| Turkey, 2003 | Cetinkaya  [24] | Cross-sectional | District | General population | RBT | 4.8 (3.5-6.1) | 1052 |
| Turkey, 2003 | Karabay  [23] | Cross-sectional | Provincial | General population | Parallel RBT and STAT | Overall: 1.3 (0.8-1.8)  Urban: 1.7 (1.0-2.4)  Rural: 1.0 (0.3-1.7) | Overall:2,098  Urban: 1,298 Rural: 800 |
| Turkey, 2005 | Vancelik  [28] | Cross-sectional | District | General population | STAT, RBT | 5.4 (3.5-7.3) (STAT);  11.9 (9.2-14.6) (RBT) | 573 |
| **Central Asia** | | | | | | | |
| Kyrgyzstan, 2006 | Bonfoh  [7] | Cross-sectional | National | General population | Parallel ELISA + RBT + Huddleson | 8.8 (4.5-16.5) | 1,774 |
| **Central and South America** | | | | | | | |
| Argentina, 2002-2005 | Marder  [29] | Routine data | District | Healthy blood donors | Huddleson | 1.4 (1.3-1.5) | 35,388 |
| Mexico, 1995-1996 | Hernández  [31] | Longitudinal | Provincial | Healthy blood donors | RBT | 2.8 (2.5-3.1) | 9590 |
