# Supplementary material for: Global Burden of Human Brucellosis: A Systematic Review of Disease Frequency
Source: PLoS Negl Trop Dis. 2012 Oct 25;6(10):e1865. doi: 10.1371/journal.pntd.0001865 (PMC3493380; doi:10.1371/journal.pntd.0001865)
Supplement: Table S2 — Selected brucellosis incidence studies by region. (DOC) [file pntd.0001865.s002.doc]

**Table S2: Selected brucellosis incidence studies by region**

| **Country,Year(s) of study** | **Author** | **Study design** | **Sampling methods** | **Study level** | **Reference population type and size** | **Diagnostic tests** | **Incidence**  **(cases per 100,000 per year)** | **Number of cases** |
| --- | --- | --- | --- | --- | --- | --- | --- | --- |
| **North Africa and Middle East** | | | | | | | | |
| Egypt  2001 | Crump  [8] | Longitudinal | Non-random purposive sampling; active case-finding | District | General population: 664,000; Acute febrile illness population: 449 | STAT and/or culture | 18.0 | 31 |
| Egypt 2002-2003 | Jennings  [9] | Longitudinal | Non-random purposive sampling; active case-finding | Provincial | General population: 2,347,249; Acute febrile illness population: 4490 | STAT and/or culture | 64.0 (2002) 70.0 (2003) | 135 (2002) 186 (2003) |
| Iran  2002-2004 | Hagh-doost [18] | Routine data | Non-random sampling; passive case-finding | District | General population: 50,000 | Serology | 141.6 | 97 |
| Iran  2006 | Hosseini  [36] | Routine data | Non-random sampling; passive case-finding | Provincial | General population | Wright Agglutination and 2ME | 13.1 | 145 |

| **Country,Year(s) of study** | **Author** | **Study design** | **Sampling methods** | **Study level** | **Reference population type and size** | **Diagnostic tests** | **Incidence**  **(cases per 100,000 per year)** | **Number of cases** |
| --- | --- | --- | --- | --- | --- | --- | --- | --- |
| Jordan 1988-1997 | Abu Sahqra  [19] | Routine data | Non-random sampling; passive case-finding | National | General population | Rose Bengal followed by STAT of positives | 16.7 (1988) 21.4 (1989) 24.1 (1990) 29.9 (1991) 22.3 (1992) 22.6 (1993) 22.6 (1994) 27.3 (1995) 26.1 (1996) 25.7 (1997) | 7,842 |
| Jordan 2003-2004 | Gargouri  [11] | Longitudinal | Non-random sampling; passive case-finding | National | General population | STAT | 130 | 31 (Sept. 2003); 9 (May 2004) |
| Palestine  1986-1996 | Awad  [10] | Longitudinal | Non-random sampling; passive case-finding | National | General population: 860,000 | RBT | 8 | 69 |
| Saudi Arabia 1997 | Elbeltagy  [16] | Routine data | Non-random sampling; passive case-finding | Provincial | General population: 40,000 | Sympto-matically or culture or paired serology or CFT | 34 | 137 |
| Saudi Arabia 1983 – 2007 | Al Tawfiq  [14] | Routine data | Non-random sampling; passive case-finding | District | Company employees: 37,000 | ELISA or STAT | 13 (1983) 33.3 (1985) 51.3 (1986) 70.7 (1987) 26.3 (1983-1992) 6 (1993-2007) | 913 |
| **Country,Year(s) of study** | **Author** | **Study design** | **Sampling methods** | **Study level** | **Reference population type and size** | **Diagnostic tests** | **Incidence**  **(cases per 100,000 per year)** | **Number of cases** |
| **Western Europe** | | | | | | | | |
| Germany  1995-2005 | Dahouk [21] | Routine data | Non-random sampling; passive case-finding | National | General population | Serology and/or culture | 0.03 (1998-2001) 0.6 (1962-1965) | 245 |
| Greece 1999-2004 | Jelasto-pulu [20] | Routine data | Non-random sampling; passive case-finding | Provincial | General population: 322,790 | Unknown | 4 | 86 |
| Greece 2002-2004 | Avdikou  [12] | Longitudinal | Non-random sampling; passive case-finding | Provincial | General population: 353,820 | ELISA or RBT or STAT | 17.3 | 152 |
| Greece 2003-2005 | Minas  [13] | Longitudinal | Non-random sampling; passive case-finding | Provincial | General population: 278,000 | ELISA, CFT, STAT, IFA or Coombs | 32.5 | 821 |
| Italy  1997-2002 | De Massis  [17] | Routine data | Non-random sampling; passive case-finding | National | General population | Unknown | 1.4 (2002)  2.7 (1990)  3.3 (1996) | - |
| **North America** | | | | | | | | |
| USA  1990-1998 | Doyle  [15] | Routine data | Non-random sampling; passive case-finding | National | General population: 322,790; Zone 1: 9,871,639; Zone 2: 45,572,965; Zone 3: 203,067,933 | Unknown | Zone 1: 0.18  Zone 2: 0.09  Zone 3: 0.02 | Zone 1: 158  Zone 2: 360  Zone 3: 398 |
